# Dental and Maxillofacial Trauma Skills Content in the Southeast Asian Dental Curricula: A Mixed Methods Study

**DOI:** 10.1111/edt.13048

**Published:** 2025-04-15

**Authors:** Rodrigo Mariño, Nabihah Dziaruddin, Kathreena Kadir, Christina P. C. Sim, Bernadette Quah, Papimon Chompu‐inwai, Varisara Sirimaharaj

**Affiliations:** ^1^ Faculty of Dentistry University of Puthisastra Phnom Pehn Cambodia; ^2^ Center for Research in Epidemiology, Economics and Oral Public Health (CIEESPO) University de la Frontera Temuco Chile; ^3^ Department of Conservative Dentistry and Oral Health Riga Stradins University Riga Latvia; ^4^ Faculty of Dentistry, Universiti Malaya Kuala Lumpur Malaysia; ^5^ Department of Restorative Dentistry, National Dental Centre Singapore Singapore; ^6^ Faculty of Dentistry National University of Singapore Singapore; ^7^ Faculty of Dentistry Chiang Mai University Chiang Mai Thailand

**Keywords:** curriculum development, dental education, dental maxillofacial trauma, mixed‐methods approach, Southeast Asia

## Abstract

**Background/Aim:**

This study aims to review contents related to dental maxillofacial trauma (DMT) in the dental curricula of Southeast Asian (SEA) countries and assess their effectiveness in equipping future dental professionals with the necessary skills for the efficient management of DMT. Additionally, the study aims to describe how the academics involved in the planning and organization of these curricula envision the incorporation of DMT within dental education.

**Methods:**

The study employed a mixed methods approach composed of two phases. Phase 1 involved a review of all printed documentation to explore the DMT curricula, including details such as the country, subject, year of study, coverage, time spent on the subject, assessment types and various training themes (e.g., management, surgical, prevention). In Phase 2, semi‐structured interviews were conducted with academics responsible for curriculum development in each school to identify how they envisaged the incorporation of DMT into the curricula.

**Results:**

Regarding curricular contents, the dataset comprised a total of 31 subjects concerning DMT contents. Findings revealed variability in the coverage of trauma education across institutions, a lack of practical training and the need for better integration and collaboration among different dental specialties. The narrative highlighted concerns about the insufficient hands‐on practice available for training in managing DMT, suggesting that students may not have enough opportunities for practical experience in this area. This deficit could potentially impact their ability to effectively manage trauma cases in a clinical setting.

**Conclusion:**

This study provides valuable insights into the current state of dental trauma education in SEA countries, identifying critical areas for improvement and potential strategies for enhancing curriculum effectiveness. It emphasizes the importance of integrating practical training and collaborative learning to better equip future dental professionals for trauma management, thereby addressing significant challenges faced by dental educators and practitioners in SEA.

## 1 Introduction

Dental and maxillofacial trauma (DMT) refers to any injury to the face or jaw caused by physical force and foreign objects [1]. DMT accounts for approximately 20% of all acute and chronic injuries globally [2], posing a substantial public health challenge due to its implications on functionality, aesthetics, psychological wellbeing and economic factors [2, 3]. In the last few decades, there has been increased recognition of the importance of the need to improve clinical competencies and knowledge around the management and treatment of dental trauma [4, 5, 6, 7]. This lack of preparedness was even highlighted as an area where more clinical experience might be required by new graduates and employers of new dental graduates [5, 8, 9, 10].

Nonetheless, for improvements to happen, it is necessary to explore essential components that should be contained in the DMT curriculum of dental programs [11]. Reports indicate that DMT in Southeast Asia (SEA) is prevalent [2, 12, 13, 14, 15, 16]. Evaluating the current state of the DMT curricula and the perspectives of those involved in the planning and execution of these programs can lead to the development of strategies to improve dental students' clinical skills and competencies in DMT. This can then translate into better treatment outcomes, as well as the incorporation of methodological strategies for DMT management training, which would respond to the educational needs of clinical competencies among dental students.

Consequently, this study aims to review the contents of the DMT dental curricula in SEA countries, specifically, the skills and competencies in the dental curricula for the management of patients with DMT and assess their effectiveness in equipping future dental professionals with the necessary skills for the efficient management of patients with DMT. Additionally, the study aims to describe how the academics involved in the planning and organizing of these curricula visualize the incorporation of DMT in the dental curricula.

This is the first study to explore the level of competence and skills required to safely handle DMT in SEA; review the challenges faced in the implementation of DMT in SEA dental curricula; and analyze academic staff's perception of the coverage of DMT in the dental curricula of dentistry programs.

## 2 Methodology

### 2.1 Study Design

To achieve the aims and objectives of this study, the project used a mixed methods approach and design. Quantitative and qualitative approaches were undertaken concurrently to effectively explore the DMT curricula. The study received institutional ethical approval from the University of Puthisastra (UP) Research Committee (Ref. no: 009IR24) and from participating other institutions (National University of Singapore IRB: NUS‐IRB‐2024‐528). The study comprised the following two main phases.

### 2.2 Phase 1

The objective of Phase 1 was to obtain information about the curricula content for the development of skills and competencies that would best prepare future dental professionals for the effective management of patients with DMT.

All courses and curricular activities officially recognized as a part of the study programs of SEA dental schools were reviewed. A comprehensive content analysis of the representation of DMT in the subjects' programs and curricular activities was carried out following guidelines on the process of analyzing documents [17]. This included:

1. The evaluation of the presence of content relating to DMT in the dental subjects and other curricular activitiestypically includes the various aspects related to dental injuries, their diagnosis, treatment and management. This evaluation was based on the following seven themes:
  - Classification and assessment of DMT.
  - Examination and diagnosis of DMT.
  - Radiographic techniques for assessment DMT.
  - Management of dental fractures, luxation injuries and avulsions.
  - Endodontic treatment of traumatized teeth.
  - Surgical management of complicated DMT cases.
  - Prevention of DMT.

The study also aimed to obtain an understanding and application of key concepts covered in the different subjects with DMT content. These contents may vary across educational institutions and programs, but provide a framework for understanding and managing DMT.

2 This assessment also included the description of the type of curriculum used, defined as:
  - Traditional: Introduction of didactic courses with a focus on basic sciences in early years and clinical sciences in later years [18].
  - Integrated: Vertical and horizontal integration of basic sciences with clinical sciences [19, 20].
  - Hybrid: Involves problem‐based learning guided by a series of lectures in any part of the curriculum [20].
  - Embedded: Hidden curriculum in which unplanned teaching messages are explicit in formal teaching [18].
3 Estimation of hours assigned to the teaching of DMT contents.
4 Evaluation of contents within each subject. This is important as when contents are known as formative only, students do not tend to review them [21].
5 Depth of coverage. Four categories of syllabus were identified [21]:
  - Consistent: Consistent syllabuses are those in which the approach to elements that allow the development of DMT in various sections of the subjects' syllabus is observed, demonstrating coherence between competencies, learning outcomes, content, methodologies and bibliography.
  - Intermediate: Intermediate syllabuses contain elements that allow the development of DMT in some sections of the subject's syllabus; however, there is no internal coherence of these elements in the subject program.
  - Emerging: Emerging syllabuses are those in which the presence of elements that allow the development of DMT is observed; however, these are not observed in all sections of the syllabus and do not follow a solid organization.
  - Not observed: when no DMT contents were found.

### 2.3 Phase 2

In this phase, academics with important roles and responsibilities for the development of the curriculum at each dental school were identified and invited to participate in a semi‐structured interview.

Prior to the commencement of the interview, the study team members obtained informed consent from the participant by providing a copy of the Plain Language Statement, verbally clarifying the aims of the study, and explaining the interview and analysis process, data security and the individual's right to withdraw. The participants then provided verbal consent.

Nine broad topics were addressed in the interviews to gain information about DMT teaching. These topics are presented in Table 1. Interviews were audio recorded (with permission from the participants) and transcribed verbatim using AvidNote software [22]. Transcriptions were reviewed by the researcher conducting the interviews and the project lead to ensure accuracy and consistency. The analysis of the interviews was carried out through thematic analysis. One researcher coded the data (RM), following the six‐phase framework outlined by Braun and Clarke [23]. This approach allowed for a systematic examination of the data, facilitating the identification of concepts and grouping them into themes relevant to the research question.

**TABLE 1:** Themes around dental and maxillofacial trauma teaching addressed in the interviews.

Assessment of justification of the decision to introduce DMT content and skills in the career curriculum
Possible follow‐up questions:
Based on your experience, what improvements or modifications would you suggest for the dental curricula to enhance the integration of DMT skills
b Identification of subjects considered by academics that incorporate DMT management skills
c Discipline and background of teaching staff
2 How do you believe these skills and competencies should be incorporated into dental curricula?
Possible follow‐up questions:
Can you provide any examples or case studies that demonstrate the successful integration of dental and DMT skills in patient management?
3 Importance attributed by the interviewee to the teaching of DMT management skills in the career curriculum and in professional performance
Possible follow‐up questions:
How do you ensure that dental students receive adequate training in DMT skills during their education?
4 How do you assess the effectiveness of the current dental curricula in preparing students to manage patients with dental and DMT needs?
5 Description of methodological strategies (e.g., activities, resources, evaluative strategies, etc.) for the management of content and DMT skills in the identified subjects
6 Are there any challenges or barriers that you have encountered in implementing DMT content and skills in the curricula?
7 Reflection on potential improvements to be made, at the micro and macro curricular level, regarding the management of DMT content and skills in the future
8 DMT reference materials and competence assessment strategies will also be analyzed
9 Are there any additional insights or perspectives you would like to share regarding the management of patients with dental and DMT needs in dental curricula?

## 3 Results

Thirteen academics from eight SEA countries were invited to participate in this study. Despite follow‐ups sent from March to August 2024, academics from four SEA countries responded to the invitation namely Cambodia, Malaysia, Singapore and Thailand.

In Cambodia, the University of Puthisastra has a 7‐year curriculum divided into 4 stages; Year 1 is the Foundation year, where basic sciences are covered; Years 2–3 cover Basic Medical and Dental Sciences, Years 4–6 are the Clinical years, and the last year, Year 7, relates to the Internship, thesis and practice management. Year 1 and Year 7 subjects were excluded from this review.

The Universiti Malaya's dental program follows a 5‐year structured curriculum. The initial 2 years (Years 1 and 2) focus on Basic Medical and Dental Sciences, covering foundational subjects such as anatomy, physiology, pathology as well as Oral Biology and Dental Materials. From Year 3 onwards, students' progress to the Clinical Phase (Years 3 to 5), where they engage in hands‐on training across various dental disciplines. The National University of Singapore (NUS) is the only dental school in the small country of Singapore and provides a four‐year curriculum. The first and second years are part of the preclinical phase of teaching, where students complete basic medical and dental science subjects. The third and fourth years make up the clinical phase, where students are exposed to clinical dental subjects and treat their own patients in comprehensive clinical sessions.

Chiang Mai University (CMU) in Thailand offers a six‐year curriculum to equip students with essential knowledge in health sciences. In the first year, students engage in General Education subjects. Years 2 and 3 emphasize Specific Courses, including Basic Sciences tailored for health science students and Basic Professional. The curriculum in years 4 to 6 provides students with practical experience and opportunities within the field.

### 3.1 Phase 1

A systematic documentary review was conducted on the study plans and subject syllabi of universities in the four dental schools. The review encompassed a total of 75 courses from Cambodia, 61 from Malaysia, 31 from Singapore and 114 from Thailand. This revealed a total of 31 subjects that incorporated at least some content related to DMT. Among these, only one course at CMU offered a dedicated subject in dental traumatology. The remaining subjects included oral and maxillofacial surgery (*n* = 8), paediatric dentistry (*n* = 5), endodontics (*n* = 4), restorative dentistry (*n* = 3), radiography (*n* = 3) and seven other related subjects. These 31 subjects were offered in the different years of the curriculum.

The overall mean duration of time allocation was 4.8 (SD 4.3)hours per subject, ranging from 1.00 to 18.50 h, with variation by country (Table 2). When excluding the course fully dedicated to DMT, the average proportion of time devoted to DMT across the other subjects was 10.5%, with a range of 1% to 33%. These variations in allocated time reflect differences in curricular emphasis and the complexity of the subject. Importantly, no significant differences were identified in the proportion of time dedicated to DMT across the countries involved in the study. When evaluated by specialty, the proportion of time dedicated to DMT was notably higher in restorative dentistry subjects (23.6%), followed by oral and maxillofacial surgery (OMS) (11.7%) and endodontics (9.7%). In paediatric dentistry and radiology, the proportions were lower, at 8.7% and 5.8%, respectively.

**TABLE 2:** Curriculum type and formal contact hours related to dental and maxillofacial trauma skill content in the Southeast Asian Doctor of Dental Surgery curricula.

Country	Year	Subject name	Curriculum type	Depth of coverage	DMT time (h)	Total subject time (h)	Type of assessment
Cambodia	2	Introduction to Dentistry	Traditional	Consistent	1.5	22.5	MCQ^a^, SA^b^
Cambodia	3	Dental Radiology	Traditional	Intermediate	3.5	30	MCQ, SA
Cambodia	3	Physio‐ Medical and Surgical Pathology	Traditional	Emerging	3	45	MCQ, SA, True or False
Cambodia	3	Community Dentistry 1 (Preventive Dentistry)	Hybrid	Intermediate	1.5	22.5	MCQ, SA
Cambodia	4	Oral Surgery II	Integrated	Intermediate	1.5	30	MCQ, SA
Cambodia	5	Endodontics 2	Traditional	Intermediate	3	22.5	MCQ, SA
Cambodia	6	Ophthalmology	Traditional	Consistent	1.5	22.5	MCQ, SA
Cambodia	6	Oral Surgery III	Hybrid	Intermediate	10.5	45	MCQ, SA
Cambodia	6	Dental Radiology 2	Traditional	Intermediate	1.5	55	MCQ, SA
Cambodia	6	Paediatric Dentistry 2	Hybrid	Consistent	6	22.5	MCQ, SA
Malaysia	3	General Surgery	Embedded	Consistent	4	235	MCQ, SA, OSCE^c^
Malaysia	3	Oral and Maxillofacial Imaging II	Integrated	Consistent	5	123	MCQ, SA, OSCE
Malaysia	3	Paediatric Dentistry I	Hybrid	Consistent	1	167	OSPE^d^
Malaysia	4	Oral and Maxillofacial Surgery II	Hybrid	Consistent	17	199	MCQ, SA
Malaysia	4	Paediatric Dentistry II	Hybrid	Consistent	9	219	MCQ, SA, OSCE
Malaysia	5	Paediatric Dentistry III	Hybrid	Consistent	2	184	MCQ, SA, OSCE
Singapore	3/4	Oral and Maxillofacial Surgery	Traditional, Integrated, Hybrid	Intermediate	10.5	46	MCQ, S, Essay
Singapore	3/4	Paediatric Dentistry	Traditional, Integrated, Hybrid	Consistent	4	25	MCQ, SA
Singapore	3/4	Endodontics	Traditional	Intermediate	2	16	SA
Thailand	3	Basic Oral Surgery	Hybrid	Intermediate	5	26	MCQ
Thailand	4	Medical Emergencies in Dental Office	Integrated	Intermediate	1	15	MCQ
Thailand	4	Practice in Oral Surgery Clinic 1	Hybrid	Intermediate	5	45	Rubric score competency: Minimum requirement
Thailand	5	Basic Oral and Maxillofacial Traumatology	Hybrid	Consistent	18.5	18.5	MCQ, Self‐assessment, Case discussion
Thailand	6	Practice in Oral Surgery Clinic 3	Hybrid	Intermediate	5	45	Rubric score, competency: Minimum requirement
Thailand	3	Basic Operative Dentistry 6	Integrated	Intermediate	5	15	MCQ
Thailand	4	Current Concept in Operative Dentistry	Integrated	Intermediate	5	15	MCQ
Thailand	5	Clinical Restorative Dentistry	Hybrid	Intermediate	5	125	Rubric score, competency: Minimum requirement
Thailand	4	Endodontics	Hybrid	Intermediate	3	33	MCQ, PBL^e^
Thailand	5	Clinical Endodontics	Hybrid	Intermediate	5	125	Rubric
Thailand	6	Seminar in Dental Emergency	Integrated	Intermediate	1	15	MCQ, SA, Assignment presentation
Thailand	6	Multidisciplinary Treatment Planning 2	Hybrid	Intermediate	3	30	MCQ, SA, Assignment presentation

^a^ MCQ: Multiple choice question.

^b^ SA: Short answer.

^c^ OSCE: objective structured clinical examinations.

^d^ OSPE: objective structured practical examinations.

^e^ PBL: Problem‐based learning.

The presence of DMT content was assessed across seven thematic areas. Tables 2 and 3 illustrate the percentage of themes covered by country, type of curriculum and depth of coverage. Four themes emerged as the most frequently addressed across the four curricula: classification and assessment of dental trauma; examination and diagnosis of dental injuries; radiographic techniques for dental trauma assessment; and management of dental fractures, luxation injuries and avulsions (Table 3).

**TABLE 3:** Curriculum themes and key concepts in dental and maxillofacial trauma in the Southeast Asian Doctor of Dental Surgery curricula.

Country	Subject name	Key concepts	Theme
Cambodia	Introduction to Dentistry	Understand the aetiology, prevention and management of dental trauma.	Classification and assessment of dental trauma. Management of dental fractures, luxation injuries and avulsions. Prevention of dental and DMT^a^.
Cambodia	Dental Radiology	Basic radiographic finding in trauma.	Examination and diagnosis of dental injuries. Radiographic techniques for DMT assessment.
Cambodia	Physio‐ Medical and Surgical Pathology	Bone and joint trauma: types of bone, fracture, types of fracture, extent of fracture. Healing process, complications in bone healing, dislocations and subluxations.	Classification and assessment of dental trauma. Examination and diagnosis of dental injuries. Management of dental fractures, luxation injuries and avulsions. Prevention of dental and DMT.
Cambodia	Community Dentistry 1 (Preventive Dentistry)	Epidemiology and causes of trauma to the orofacial region. Signs and symptoms. Prevention of injuries e.g., from sports, car accidents, moto/bicycle accidents, assaults, child abuse. Case studies on child abuse.	Classification and assessment of dental trauma. Examination and diagnosis of dental injuries. Prevention of dental and DMT.
Cambodia	Oral Surgery II	Crown fracture or luxation of adjacent tooth. Soft tissue injuries and hard tissue fractures. Hemorrhage, emphysema and nerve injury. Mandibular fracture and TMJ dislocation. Oroantral communication and tooth displacement into maxillary sinus.	Examination and diagnosis of dental injuries. Management of dental fractures, luxation injuries and avulsions. Surgical management of complicated DMT cases.
Cambodia	Endodontics 2	2018 IADT Guidelines. Diagnosis and management of pulpal injuries. Loss of vitality and other complications following dental injuries. Which injuries are most damaging to the pulp?. Signs of pulpal pathology.	Classification and assessment of dental trauma. Examination and diagnosis of dental injuries. Management of dental fractures, luxation injuries and avulsions. Endodontic treatment of traumatized teeth.
Cambodia	Ophthalmology	Injuries to the eye—blunt and penetrating trauma. Injuries to the eye socket. Blow‐out fractures. Corneal foreign body.	Classification and assessment of dental trauma. Examination and diagnosis of dental injuries. Management of dental fractures, luxation injuries and avulsions. Prevention of DMT.
Cambodia	Oral Surgery III	Epidemiology of OMF trauma. Causes of OMF trauma. Types of OMF injuries. First aid—review of BLS. Assessment. Principles of management. Mandibular fractures. Condylar fractures. Middle third fractures (Le Fort fractures). ZMC fracture. Naso‐Orbito‐Ethmoidal fractures. –classification –causes –presentation –management –complications	Classification and assessment of dental trauma. Examination and diagnosis of dental injuries. Radiographic techniques for DMT assessment. Management of dental fractures, luxation injuries and avulsions. Surgical management of complicated DMT cases. Prevention of dental and DMT.
Cambodia	Dental Radiology 2	Radiographic appearance of dentoalveolar and maxillofacial trauma. Displacement injuries. Fractures. Resorption following trauma. Types of imaging for dentoalveolar and maxillofacial trauma. Standard radiographic views for different types of maxillofacial trauma. Other useful imaging techniques and Radiographic diagnosis of maxillofacial trauma.	Examination and diagnosis of dental injuries. Radiographic techniques for DMT assessment.
Cambodia	Paediatric Dentistry 2	Epidemiology of trauma. Classification of trauma. History and examination. The first visit. Special tests. Guidelines to management (IADT). Soft tissue injuries. Dental injuries. Enamel fractures. Uncomplicated enamel dentine fractures. Complicated enamel dentine fractures. Root fractures. Crown root fractures. Displacement injuries. Concussion and subluxation. Lateral displacement. Intrusion. Extrusion. Avulsion. Complications of injuries and their management. Root resorption. Epidemiology of child abuse. Signs of child abuse. What should the dentist do if they suspect child abuse?. Reporting child abuse.	Classification and assessment of dental trauma. Examination and diagnosis of dental injuries. Radiographic techniques for DMT assessment. Management of dental fractures, luxation injuries and avulsions. Prevention of dental and DMT.
Malaysia	General Surgery	Understanding the different types of fractures. Categorizing fractures based on their characteristics. The biological process of bone healing. Management strategies for different types of fractures Treatment protocols to ensure proper healing and recovery. Techniques and tools for maintaining and securing an airway in patients. Emergency procedures for airway obstruction. Advanced airway management strategies in surgical and trauma settings. Hands‐on training in performing CPR. Understanding the latest guidelines and protocols for effective resuscitation.	Classification and assessment of dental trauma. Examination and diagnosis of dental injuries. Radiographic techniques for DMT assessment. Management of dental fractures, luxation injuries and avulsions. Surgical management of complicated DMT cases.
Malaysia	Oral and Maxillofacial Imaging II	Recognizing radiographic signs/appearance of trauma, such as discontinuity in bone structure, abnormal tooth positioning and radiolucency indicating fractures. Types of imaging for dentoalveolar and maxillofacial trauma. Other useful imaging techniques and radiographic diagnosis of maxillofacial trauma. Techniques for intraoral radiographs. Positioning and angulation.	Classification and assessment of dental trauma. Examination and diagnosis of dental injuries. Radiographic techniques for DMT assessment.
Malaysia	Paediatric Dentistry I	Understand that composite wire to stabilize injured teeth after dental trauma. Recognize the types of injuries that require splinting, such as luxation, subluxation and avulsion.	Management of dental fractures, luxation injuries and avulsions.
Malaysia	Oral and Maxillofacial Surgery II	Understanding the initial steps in the management of maxillofacial trauma. Learning the protocols for immediate care and stabilization of patients with maxillofacial injuries. Cranial nerve assessment. Classification of different types of facial soft tissue injuries. Recognizing the clinical features associated with facial soft tissue injuries. Investigating the appropriate diagnostic methods. Managing the treatment and care of patients with facial soft tissue injuries. Understanding the potential complications arising from facial soft tissue injuries. Learn the classification, causes, clinical features, diagnostic, investigations, management strategies and potential complications of dentoalveolar injuries. Comprehensive patient assessment—systematic approach to history taking. Clinical examination and investigations. Differential diagnosis. Documentation and record keeping. Overview of different wiring methods used in maxillofacial trauma: e.g., arch bars, intermaxillary fixation, eyelet wiring. Tools and materials used in wiring procedures. Step by step techniques. Strategies for managing and troubleshooting complications. Instructions for postoperative care and wiring maintenance.	Classification and assessment of dental trauma. Examination and diagnosis of dental injuries. Radiographic techniques for DMT assessment. Management of dental fractures, luxation injuries and avulsions. Surgical management of complicated DMT cases. Prevention of dental and DMT.
Malaysia	Paediatric Dentistry II	Classification of trauma. Epidemiology of trauma. History and examination. Investigation—special tests. Principle of trauma management. Guidelines to management (IADT). Soft tissue injuries. Dental injuries. Enamel fractures. Uncomplicated and complicated enamel dentine fractures. Root fractures. Crown root fractures. Concussion and subluxation. Lateral displacement. Intrusion. Extrusion. Avulsion. Complications of injuries and their management. Root resorption. Healing. Prevention. Understanding immature teeth anatomy and development. Clinical and radiographic evaluation to determine the extent of damage and stage of root development. Treatment options for vital and non‐vital teeth in immature anterior permanent teeth.	Classification and assessment of dental trauma. Examination and diagnosis of dental injuries. Radiographic techniques for DMT assessment. Management of dental fractures, luxation injuries and avulsions. Endodontic treatment of traumatized teeth. Surgical management of complicated DMT cases. Prevention of dental and DMT.
Malaysia	Paediatric Dentistry III	Small group activities—to develop treatment plans for various trauma cases. Groups present their treatment plans.	Classification and assessment of dental trauma. Examination and diagnosis of dental injuries. Radiographic techniques for DMT assessment. Management of dental fractures, luxation injuries and avulsions. Endodontic treatment of traumatized teeth. Surgical management of complicated DMT cases. Prevention of dental and DMT.
Singapore	Oral and Maxillofacial Surgery	Maxillofacial injuries: Diagnosis to treatment planning. Maxillofacial injuries: Mandible fractures. Middle third fractures. Wiring Technique. Understand the examination, diagnosis and treatment options for patients with maxillofacial injuries.	Surgical management of complicated DMT cases. Covered but for DMT. NOT specific to just dental trauma. Classification and assessment of trauma. Examination and diagnosis of injuries. Radiographic techniques for trauma assessment.
Singapore	Paediatric Dentistry	Traumatic injuries to anterior teeth‐I. Traumatic injuries to anterior teeth‐II. Trauma case series. Clinical evaluation, diagnosis and management of dental trauma in children.	Classification and assessment of dental trauma. Examination and diagnosis of dental injuries. Radiographic techniques for DMT assessment. Management of dental fractures, luxation injuries and avulsions. Endodontic treatment of traumatized teeth.
Singapore	Endodontics	Aetiology, classification and management of traumatic dental injuries.	Classification and assessment of dental trauma. Examination and diagnosis of dental injuries. Management of dental fractures, luxation injuries and avulsions. Endodontic treatment of traumatized teeth.
Thailand	Basic Oral Surgery	Principle of oral surgery. Inflammation and wound healing. Hemostasis. Armamentarium in oral surgery. Flap in oral surgery. Suture techniques and suture materials. Exodontia and management of complication. Tooth transplantation. Analgesic drugs and antibiotics in dentistry.	Classification and assessment of dental trauma. Examination and diagnosis of dental injuries. Radiographic techniques for DMT assessment. Management of dental fractures, luxation injuries and avulsions. Surgical management of complicated DMT cases.
Thailand	Medical Emergencies in Dental Office	Common medical emergencies in dental office: unconsciousness, respiratory distress, altered consciousness, chest pain, seizure, drug overdose and allergy. Aetiology, signs and symptoms, differential diagnosis and management, basic cardiopulmonary resuscitation, principles of prevention and preparation for medical emergencies in dental office and used of emergency kits.	Classification and assessment of dental trauma. Examination and diagnosis of dental injuries.
Thailand	Practice in Oral Surgery Clinic 1	Basic clinical practice in oral surgery including simple exodontia, management of post‐ extraction complications and wound care, practice as dental assistant in minor oral surgery.	Classification and assessment of dental trauma. Examination and diagnosis of dental injuries. Management of dental fractures, luxation injuries and avulsions. Surgical management of complicated DMT cases.
Thailand	Basic Oral and Maxillofacial Traumatology	Principles of oral and maxillofacial traumatology. Clinical and radiographic examination and diagnosis. Management of dentoalveolar injury including both primary and permanent teeth and fracture of facial bone. Correction of posttraumatic malocclusion, malunion and non‐union.	Classification and assessment of dental trauma. Examination and diagnosis of dental injuries. Radiographic techniques for DMT assessment. Management of dental fractures, luxation injuries and avulsions. Endodontic treatment of traumatized teeth. Surgical management of complicated DMT cases.
Thailand	Practice in Oral Surgery Clinic 3	Observation in surgical management of oral and maxillofacial fractures, dentofacial deformity and general anaesthesia procedure in operating theatre.	Classification and assessment of dental trauma. Examination and diagnosis of dental injuries. Radiographic techniques for DMT assessment. Management of dental fractures, luxation injuries and avulsions. Surgical management of complicated DMT cases.
Thailand	Basic Operative Dentistry 6	Advanced resin composite restorative treatment in complicated case. Properties and clinical usage of Glass Ionomer Cement (GIC) and Resin Modified Glass Ionomer Cement (RMGI). Liners and bases. Treatment planning in complicated patients. Biological aspects in operative procedure.	Classification and assessment of dental trauma. Examination and diagnosis of dental injuries. Radiographic techniques for DMT assessment. Management of dental fractures, luxation injuries and avulsions.
Thailand	Current Concept in Operative Dentistry	Contemporary concepts of dental adhesive system in operative dentistry. Restorative treatment in anterior with adhesive material, tooth bleaching and enamel micro abrasion, restorative treatment in complex badly broken‐down teeth and failure in restorative treatment.	Classification and assessment of dental trauma. Examination and diagnosis of dental injuries. Radiographic techniques for DMT assessment. Management of dental fractures, luxation injuries and avulsions.
Thailand	Clinical Restorative Dentistry	Clinical practice in complicated operative procedures including restoration of proximal surface involving incisal edge of anterior teeth. Clinical practice of either simple restoration of endodontically‐treated tooth with post and core or crown construction for vital teeth in patients.	Classification and assessment of dental trauma. Examination and diagnosis of dental injuries. Radiographic techniques for DMT assessment. Management of dental fractures, luxation injuries and avulsions.
Thailand	Endodontics	Basic sciences of pulp and periapical tissues. Diseases and pathology of pulp and periapical tissue. Examination, diagnosis and endodontic treatment planning. Principles and procedures of endodontic treatment. Evaluation of treatment outcome, retreatment and endodontic surgery.	Classification and assessment of dental trauma. Examination and diagnosis of dental injuries. Radiographic techniques for DMT assessment. Endodontic treatment of traumatized teeth.
Thailand	Clinical Endodontics	Clinical practice in endodontics including. Diagnosis of pulpal and periapical diseases. Practice uncomplicated root canal treatment (anterior teeth). Evaluation of treatment outcome. Retreatment of failed cases.	Classification and assessment of dental trauma. Examination and diagnosis of dental injuries. Radiographic techniques for DMT assessment. Endodontic treatment of traumatized teeth.
Thailand	Seminar in Dental Emergency	Seminar in management of dental emergencies emphasizing relief of pain, minimizing complications and preventing tooth loss and also making the referral of the patient for proper dental treatment.	Classification and assessment of dental trauma. Examination and diagnosis of dental injuries. Management of dental fractures, luxation injuries and avulsions. Endodontic treatment of traumatized teeth. Surgical management of complicated DMT cases.
Thailand	Multidisciplinary Treatment Planning 2	Practice in dental treatment planning for complicated cases by integrating knowledge on oral diagnostic science, diagnosis, oral surgery, restorative dentistry, periodontology, prosthetics, orthodontics, paediatric dentistry and preventive dentistry.	Classification and assessment of dental trauma. Examination and diagnosis of dental injuries. Management of dental fractures, luxation injuries and avulsions. Endodontic treatment of traumatized teeth. Surgical management of complicated DMT cases.

^a^ DMT: Dental and maxillofacial trauma.

Key concepts covered by the curriculum (Table 3) include an overview of DMT's aetiology, prevention and management, as well as comprehensive coverage of trauma types. It highlights the epidemiology of trauma, including child abuse and sports injuries. Key concepts included a reliance on the 2018 IADT Guidelines [24, 25, 26, 27], a commitment to evidence‐based practices, and underlined the importance of preventive strategies for at‐risk groups. Additionally, emphasis on radiographic techniques underscores the need for accurate diagnosis, while detailed management strategies reflect a structured treatment approach, including advanced techniques. The inclusion of educational components, such as case studies and hands‐on training, enhances practitioner preparedness. However, potential discrepancies in regional resource availability and a disproportionate focus on certain injuries may require attention, as less common trauma types also need proper consideration.

In the UP's Doctor in Dental Surgery curriculum encompasses ten subjects incorporate DMT training content; specifically, four subjects from Years 2 to 3 and six subjects from Years 4 to 6. The total duration allocated for DMT content across these subjects amounts to 32 h, constituting approximately 10.8% of the overall hours assigned to these courses. The distribution of DMT content hours exhibited variability, ranging from 7.2% in Dental Radiology II to 26.7% in Paediatric dentistry II, both of which are offered in Year 6 (Table 2).

The DMT curriculum at UP primarily adheres to a traditional educational model, with an overall depth of coverage classified as ‘Intermediate’ (60%). Additionally, there are three subjects in which the level of coverage is categorized as ‘Consistent’ and in one as ‘Emerging’. Assessment methods for DMT content predominantly rely on written examinations, including multiple‐choice questions (MCQs) and short‐answer questions. The focus is largely on examination and diagnosis, classification and management of DMF, with a secondary emphasis on the prevention of such injuries. However, the curriculum provides comparatively limited coverage of radiographic techniques, surgical management of dental traumatic injuries and particularly endodontic treatment for traumatized teeth (Table 4).

**TABLE 4:** Dental and maxillo‐facial trauma‐related themes in Southeast Asian countries.

Maxillo‐facial trauma theme	Cambodia %	Malaysia %	Singapore %	Thailand %	Total %
Classification and assessment of dental trauma	70.0	83.3	100.0	100.0	87.1
Examination and diagnosis of dental injuries	90.0	83.3	100.0	91.7	90.3
Radiographic techniques for dental trauma assessment	40.0	83.3	66.7	66.7	61.3
Management of dental fractures, luxation injuries and avulsions	70.0	83.3	66.7	75.0	74.2
Endodontic treatment of traumatized teeth	10.0	33.3	66.7	41.7	32.3
Surgical management of complicated dental trauma cases	20.0	66.7	33.3	50.0	41.9
Prevention of dental and maxillo‐facial trauma	60.0	50.0	0.0	0.0	29.0

In Malaysia, the Universiti Malaya's curriculum for DMT training comprised six subjects, distributed across 3 years. Trauma management is incorporated beginning in Year 3 (three subjects), initially through theoretical instruction. Practical training and case‐based discussion intensify in Years 4 (two subjects) and one subject in Year 5, where students manage trauma cases under clinical supervision. Collectively, these subjects account for a total of 34 h, representing approximately 3.35% of the total hours allocated to the respective subjects. The percentage of hours dedicated to DMT varied across subjects, ranging from 1% in Paediatric Dentistry I in Years 3 and 5 to 9% in Oral Surgery II in Year 4 (Table 2).

The Malaysian DMT curriculum predominantly employs a hybrid model, accounting for 66.7% of the curriculum content and demonstrating an overall ‘consistent’ depth of coverage. Assessment methods for DMT content included MCQs, short‐answer questions, and objective structured clinical examinations (OSCEs) and objective structured practical examinations (OSPE), which collectively comprised 83.3% of the evaluative components.

The focus of the DMT curriculum in Universiti Malaya encompasses the examination and diagnosis, classification, radiographic techniques and management of dental traumatic injuries, with relatively less emphasis placed on the surgical management and prevention of such injuries. Notably, the aspects addressing endodontic treatment for traumatized teeth received less coverage, constituting only 33.3% of the curriculum (Table 2).

The instruction of DMT in Singapore is covered within three subjects, all of which are in the clinical phase of the program (Years 3–4). Collectively, these subjects account for a total of 16.5 h, representing an average of 12.6% (range of 10.8% to 13.5%) 0 of the total hours assigned to those courses. The DMT curriculum adopts a predominantly hybrid model (66.7%) and exhibits an ‘Intermediate’ depth of coverage (66.7%). The assessment of DMT content is primarily conducted through MCQs and short‐answer formats, which together comprise 66.7% of the evaluative methods. One subject, Oral and Maxillofacial Surgery, incorporates essays as an additional form of assessment alongside MCQs and short answers.

The themes covered in the DMT curriculum include examination and diagnosis, classification, and prevention of dental traumatic injuries, with comparatively less emphasis placed on the surgical management of such injuries, and no inclusion of content addressing the prevention of dental traumatic injuries (Table 4).

At CMU in Thailand, twelve subjects featured DMT content, which comprises approximately 61.5 h dedicated to teaching these contents across Years 2 to 6 (Table 2). The DMT curriculum at CMU predominantly adopts a hybrid approach (66.7%), with four subjects employing an integrated model. Assessments of DMT competency are exclusively conducted through written examinations, including both MCQs and short‐answer questions. Moreover, certain subjects incorporate assessments designed to meet minimum competency requirements (25%).

The CMU curriculum places an emphasis on classification and assessment, examination and diagnosis, radiographic techniques and the management of DMT, with lesser focus provided to the surgical management of dental traumatic injuries. Aspects concerning endodontic treatment of traumatized teeth have comparatively less coverage at CMU (41.7%), and there is no documented inclusion of content addressing the prevention of dental traumatic injuries (Table 4).

### 3.2 Phase 2: Information Retrieved From the Interviews With Academic Staff

A total of eight academics participated in this phase of the study (one from Cambodia; two from Malaysia, one from Singapore and four from Thailand). These informants provided insights regarding primary themes, as shown in Table 1. Furthermore, the analysis revealed five emerging themes related to barriers encountered in the educational context.

#### 3.2.1 DMT In the Undergraduate Curriculum Coverage

The interviewees generally revealed the presence of DMT contents in the undergraduate (UG) curricula of the four countries. This comprehensive coverage of DMT topics in the curricula provided students with a comprehensive theoretical understanding of DMT topics, where various aspects such as airway management, trauma classification and treatment options for DMT are addressed, although at different levels of coverage at the different dental schools participating in this study.

‘The quality of trauma teaching for maxillofacial trauma at XX is quite acceptable, quite good’.

#### 3.2.2 Barriers to DMT Training

Interviewees expanded on barriers to the inclusion of DMT content into the dental curricula. These could be grouped into:

##### 3.2.2.1 Insufficient Hands‐On Practice in DMT Training

Interviewees unanimously advocated a dedicated emphasis on DMT within the curriculum to ensure that students acquire the necessary skills and knowledge to competently manage such cases in their future practice. In particular, they underscored concerns regarding inadequate opportunities for hands‐on practice for these DMT topics.

‘Hands‐on training … should be added so that the student can have more exposure’.

‘In our dental course, the training for maxillofacial trauma (DMT) is mostly theoretical, with lectures, case discussions and seminars, but without enough hands‐on practice’.

They highlighted a disproportionate focus on theoretical contents over practical exposure and the application of skills necessary for effectively managing DMT, which limits their ability to gain practical experience, affecting their proficiency in addressing trauma cases within a clinical environment.

##### 3.2.2.2 Shortage of DMT Patients

Another challenge highlighted by the informants in providing more hands‐on experience for managing DMT includes the scarcity of trauma patients in dental school clinics, which restricts students' opportunities for real‐life management of DMT patients, essential for developing their clinical skills.

‘The main problem is about the quantities of the patients …’

##### 3.2.2.3 Lack of Integration Within Dental Education

Participants also emphasized the need for curriculum development to enhance a more integrated DMT educational experience. Firstly, incorporating DMT skills into different subjects and courses can provide a way for students to attain a balanced understanding of DMT management and enhance their clinical competencies. Additionally, by addressing overlapping content and integrating various specialties, educators can create a more streamlined and effective learning environment for students.

‘Integration and collaboration among different dental specialties are needed to ensure comprehensive coverage of trauma’.

‘… we don't have enough collaboration between interdisciplinary to discuss and plan the teaching methods before the course start’.

#### 3.2.3 Teaching Techniques

Related to this point of insufficient training, the use of simulation technologies and partnerships with hospitals for practical training was suggested as effective strategies to enhance practical training in DMT management. Interviewees suggested that DMT contents could be taught and learned through direct observation of clinicians in postgraduate programs treating DMT patients in a clinical setting. However, it is important to note that this alternative would only be possible in schools that have postgraduate courses covering DMT.

‘The use of case discussions and interdisciplinary collaboration as effective teaching methods for DMT skills’.

The discussions included the importance of varied teaching methods to keep students engaged. This includes hybrid approaches that combines traditional lectures with interactive elements such as case‐based learning (CBL) and seminars and simulations [28]. Simulation allows students to practice skills in a controlled setting, while collaborations with hospitals can facilitate opportunities for real‐world exposure and learning experiences.

‘… although it's not a real one, it's a simulated one. But at least they have and I'm not sure whether if we can Increased number of CBL if that will be helpful as well, just to increase the exposure to them’.

Other suggestions were for the incorporation of digital tools and live surgeries into dental education to enhance the students' learning experience and provide them with practical exposure on the management of DMT.

#### 3.2.4 Integration of Collaborative Learning Experiences With Other Medical Disciplines

Interviewees highlighted the importance of integration within the dental curriculum and of interdisciplinary collaboration in DMT education. They indicated thatcollaborative learning experiences with other health disciplinescan improve students' understanding of DMT management and, at the same time, develop essential teamwork skills among various healthcare professionals, foster teamwork in patient care and enhance overall educational outcomes.

‘We need to have a subject alignment with all the disciplines and consolidate or integrate some of the topics’.

#### 3.2.5 Assessment Strategies

The absence of evaluation specific to DMT was acknowledged. Moreover, interviewees emphasized the importance of assessing not only theoretical knowledge but also practical decision‐making skills that are critical in real‐world clinical situations. Particularly, to implement the assessment of students' competencies in managing DMT. They emphasized the use of the various assessment strategies employed in dental education (i.e., written examinations, portfolio of case studies, reflective writings, self‐assessments of their progress over time, case discussion activities, etc.) to better evaluate students' readiness to handle DMT cases effectively and ensure that students can make swift and informed decisions in clinical settings. Interviewees also put an emphasis on the need to evaluate students' attitudes towards DMT training.

#### 3.2.6 The Way Forward

To further address the challenges identified in this study, several recommendations were proposed. First, regarding plans for the improvement of DMT content within the undergraduate curricula, academics mentioned the challenge of focussing on training students about the management of medically complex trauma cases.

‘The feedback that we often get is that they are usually unable to manage it as a new dental graduate’.

Along the same line, interviewees recognized the increasing number of trauma older patients and the need for appropriate management [29]. Additionally, they highlighted the need for further research on the current state of DMT education in SEA dental schools.

## 4 Discussion

The findings from this review provide insight into the current state of education in DMT across four countries: Cambodia, Malaysia, Singapore and Thailand. By analysing 31 courses that include DMT content, significant variations emerged in curricular emphasis, depth of coverage and assessment methodologies. Nonetheless, DMT content usually was in subjects in the later years of the courses.

Only one dedicated course on dental traumatology was identified in Thailand. In the other participating countries, the majority of DMT content is integrated within broader subjects such as oral and maxillofacial surgery and paediatric dentistry. Furthermore, Thailand also demonstrated a more integrated educational approach, as evidenced by a higher number of subjects encompassing DMT content and a greater total number of instructional hours allocated to the teaching of DMT.

The analysis of course content reveals a strong emphasis on oral and maxillofacial surgical disciplines followed by paediatric dentistry and endodontics. This supports the conclusions of Berlin and Levin about the challenges of dental trauma education [10], but contrasts with O'Connell and Olegário [28] who reported that most contents were in paediatric dentistry subjects, followed by endodontics. They also reported that 22.5% of participating countries had a separate course in DMT, which is consistent with our finding of 1 in four (25%) [28].

The average time allocated to DMT subjects across all participating countries was approximately 4.6 h, with individual course durations ranging from 1 to 18.5 h. This is lower than the 6–20 h reported in O'Connell and Olegário's review [28]. However, the proportion of time dedicated to DMT within each subject varied considerably, with a significant emphasis on restorative dentistry. Given that DMT can occur at any life stage, reports have indicated higher incidence rates during infancy, childhood and adolescence [30]. Nevertheless, the allocation of time to DMT in paediatric dentistry was lower than one might anticipate, standing at only 8.7%.

Although the curricula across various institutions may appear comprehensive, limited exposure to real‐world trauma cases and a lack of structured practical training inhibit students' preparedness to effectively manage DMT [10, 11, 28, 31]. The review identified four predominant themes that consistently appear across the curricula: ‘Classification and assessment of dental trauma’; ‘Examination and diagnosis of dental injuries’; ‘Radiographic techniques for trauma assessment’; and ‘Management of dental fractures, luxation injuries, and avulsions’. However, areas such as ‘Prevention of DMT’, ‘Endodontic treatment of traumatized teeth’ and the ‘Surgical management of complicated DMT cases’ were less frequently covered. While this suggests a consensus on essential competencies for dental students, they also raise concerns regarding graduates' preparedness to manage such situations effectively. Notably, continuing professional development (CPD) short courses and conferences have been organized in these countries to cover some of these contents [32, 33, 34, 35].

Interviews conducted as part of this study confirmed the distinct variations in curriculum coverage and opportunities for practical, hands‐on training among dental schools in SEA. They highlighted the gap between theoretical knowledge and practical skills, which may present challenges for graduates as they transition into professional practice [26]. Interviewees underscored the necessity for educational strategies with emphasis on clinical exposure, decision‐making and clinical reasoning within a controlled environment, thereby enhancing the confidence and competence of students. Additionally, they recognized the need for improved integration and collaboration among different dental specialties to ensure comprehensive coverage of the subject matter while avoiding both unnecessary repetition and fragmentation of the information [11].

In terms of assessment methods employed across the countries, MCQs and short answer questions predominated. While these methods can effectively evaluate knowledge acquisition, they may not adequately assess the practical skills and clinical reasoning essential for the effective management of DMT and students' readiness for real‐world clinical situations. Only two countries reported the implementation of more comprehensive assessments of clinical competencies, such as objective structured clinical examinations (OSCEs) or practical assessments.

While this study offers valuable insights into the training of DMT among dental schools in SEA, it is important to acknowledge its limitations. The most significant limitation lies in the lack of generalizability of findings to other dental programs within the region; notably, the findings may not even hold within the participating countries, except for Singapore, which has only one dental school. Variability in the instruction of DMT competencies is anticipated across different institutions, which may further impact the overall findings.

Despite these limitations, we believe that the methodology employed in this study facilitates the identification of preliminary insights that can inform future research and educational initiatives. The adoption of a mixed‐methods design represents a key strength of this study, as it provides a more comprehensive understanding of the research problem than could be achieved using either quantitative or qualitative approaches alone. By blending both methodologies, this design mitigates the limitations inherent to each, thereby enriching the findings.

Moving forward, future research should involve longitudinal and qualitative studies to track the career progression of graduates and their ability to manage DMT cases in a professional practice context, thereby identifying areas for improvement. This information would provide valuable insights into graduates' competency in handling DMT, as well as the real‐world applicability of their DMT training, informing subsequent curriculum adjustments, enhancing preparedness for their profession's demands.

Additionally, new cohorts of dental students may have different needs and learning approaches, and challenges to achieve educational goals related to dental trauma than in the past [10]. For example, Artificial Intelligence (AI) research is a rapidly advancing field capable of performing tasks traditionally requiring human cognition. In the context of dental trauma, AI research encompasses various subfields, including machine learning, natural language processing, computer vision, robotics and reinforcement learning [10, 35, 36, 37].

The insights from this analysis not only highlight the challenges faced by dental educators but also present opportunities for growth and improvement in the field of DMT training. By addressing these gaps through teaching initiatives, fostering collaboration among specialties and implementing targeted research initiatives, dental schools can enhance their curricula and ultimately improve graduates' preparedness to manage maxillofacial trauma effectively.

Additionally, the findings of this study provide insights into the exposure of dental students across various subjects. While the current state of DMT education in dental schools in SEA demonstrates a focus on theoretical knowledge, practical training and clinical exposure remain areas requiring attention. The analysis of coverage, time allocation and assessment types reveals areas for enhancement in dental education. By addressing these factors, educational institutions can improve training quality and better prepare students for their future roles as competent dental professionals.

## Author Contributions

R.M. conceived the idea; R.M., N.D., K.K., C.P.C.S., B.Q., P.C. and V.S. collected the data; R.M. analysed the data; and R.M. led the writing.

## Conflicts of Interest

The authors declare no conflicts of interest.

## Data Availability

Data sharing not applicable to this article as no datasets were generated or analysed during the current study.
